# Supplementary material for: Predictors of Mental Health Literacy among Parents, Guardians, and Teachers of Adolescents in West Malaysia
Source: Int J Environ Res Public Health. 2023 Jan 1;20(1):825. doi: 10.3390/ijerph20010825 (PMC9819300; doi:10.3390/ijerph20010825)
Supplement: Supplementary file 1 [file ijerph-20-00825-s001.zip › Table S1 Interaction.pdf]

Table S2. Interaction terms in the preliminary main effect model.

Interaction Terms	Unstandardized B	Coefficients Std. Error	Standardized Coefficients Beta	t	Sig.	95.0% Confidence Interval for B	
						Lower Bound	Upper Bound
Contact_income	−0.324	0.529	−0.061	−0.613	0.540	−1.362	0.714
Contact_age	−0.017	0.040	−0.069	−0.418	0.676	−0.095	0.061
Contact_training	−0.040	1.007	−0.002	−0.040	0.968	−2.017	1.937
Income_age	−0.006	0.026	−0.050	−0.211	0.833	−0.057	0.046
Income_training	0.792	0.700	0.109	1.131	0.259	−0.583	2.167
Age_training	−0.003	0.046	−0.008	−0.056	0.956	−0.093	0.088

note: significance value of >0.50 indicates no significant interaction between the two variables.
