# Supplementary material for: Predictors of Mental Health Literacy among Parents, Guardians, and Teachers of Adolescents in West Malaysia
Source: Int J Environ Res Public Health. 2023 Jan 1;20(1):825. doi: 10.3390/ijerph20010825 (PMC9819300; doi:10.3390/ijerph20010825)
Supplement: Supplementary file 1 [file ijerph-20-00825-s001.zip › Table S2 Multicollinearity.pdf]

Table S2. Assessment of multicollinearity in the model.

Model	Unstandard-ized B	Coefficients Std. Error	Standardized Coefficients Beta	t	Sig.	95.0% confidence interval for B		Collinearity Statistics	
						Lower bound	Upper bound	Tolerance	VIF
(Constant)	44.025	0.754		58.382	0.000	42.545	45.506		
Contact	1.388	0.353	0.132	3.936	0.000	0.696	2.080	0.982	1.019
Income	0.680	0.202	0.116	3.365	0.001	0.283	1.076	0.934	1.071
Age	-0.040	0.017	-0.082	-2.371	0.018	-0.073	-0.007	0.932	1.073
Training	1.019	0.451	0.076	2.259	0.024	0.134	1.904	0.989	1.011

note: the VIF (variance-inflation factor) measures the extent of multicollinearity problem. VIF of <2.5 shows no multicollinearity problem in this model.
